# Tuning *Dunaliella tertiolecta* for Enhanced Antioxidant Production by Modification of Culture Conditions

**DOI:** 10.1007/s10126-021-10041-8

**Published:** 2021-06-30

**Authors:** Uttam K. Roy, Birthe V. Nielsen, John J. Milledge

**Affiliations:** 1 School of Architecture, Building and Civil Engineering, Loughborough University, Epinal Way Leicestershire, Loughborough, LE11 3TU United Kingdom; 2 Algae Biotechnology Research Group, Faculty of Engineering and Science, University of Greenwich, Central Avenue, Chatham Maritime, Kent, ME4 4TB England United Kingdom

**Keywords:** Microalgae, *Dunaliella tertiolecta*, Culture conditions, Antioxidants, Enzyme activity

## Abstract

Microalgae, a popular source of food and bioactive compounds, accumulate antioxidants in response to culture condition stresses. Using a factorial design (3 × 3), the effect of light, temperature, and nitrogen level on chlorophyll and carotenoids, total protein, total phenolic, ascorbate and glutathione content, and enzyme (catalase (CAT), superoxide dismutase (SOD), and peroxidase (POD)) activities in *Dunaliella tertiolecta* was studied. Data were analysed using Design of Experiments (DoE), and recommendations are made for optimum cultivation conditions to achieve the highest antioxidant content (phenolics, ascorbate and glutathione) or enzyme (CAT, SOD, and POD) activities. This is the first study to apply three levels of three factors during cultivation to tune *Dunaliella tertiolecta* for optimal antioxidant production.

## Introduction

Subjected to oxidative stress, *Dunaliella* accumulates different biomolecules, including antioxidants, and it has been suggested that *D. salina* could be a useful source for enzyme production (Ben-Amotz and Avron 1990; El-Baky et al. 2004). Microalgae can be used as bio-factories for large-scale production of cellulase, galactosidases, phytase, lipase amylase, and antioxidant enzymes which are commercially used in food, animal feed, textile, biofuel, and in the chemical industry (Brasil et al. 2017; Georgianna et al. 2013).

Antioxidants accumulate to eliminate oxidative stress, which occurs due to the generation of endogenous reactive oxygen species (ROS) during cell metabolism (photosynthetic reaction or photorespiration) (Gill and Tuteja 2010). Microalgae have developed an antioxidant defence system to alleviate the adverse effect of ROS which is composed of superoxide dismutase (SOD), catalase (CAT), ascorbate peroxidase (APX), glutathione reductase (GR), monodehydroascorbate reductase (MDHAR), dehydroascorbate reductase (DHAR), and (glutathione peroxidase (GPX) as well as non-enzymatic molecules (ascorbate, glutathione, carotenoid, phenolic, tocopherol, and proline) (Al-Rashed et al. 2016; Ambati et al. 2019; Cirulis et al. 2013; Michalak 2006; Nimse and Pal 2015; Rahal et al. 2014; Şen 2012; Sharma et al. 2012). Research has shown that *Dunaliella* strains exposed to challenging culture conditions could cause extensive intracellular ROS production, which would enhance antioxidant responses (Benavente-valdes et al. 2016; Juneja et al. 2013). Culture conditions include light or spectral variation of light, nutrient availabilities, temperature, heavy metals, pH, chemical ingredients, and is usually a combination of several of these (Al-Rashed et al. 2016; Haghjou et al. 2009; Janknegt et al. 2009; Lv et al. 2016; Tian and Yu 2009).

Culture conditions, such as light irradiation (intensity, ultraviolet (UV) irradiation, wavelength, colour), variation of temperature, nitrogen levels (starvation or limitation) affect the cell’s chloroplastic protein synthesis, the rate of photosynthesis, oxidative phosphorylation in cells, and any interruption of these cellular metabolisms can induce oxidative stress, which is then mitigated by the accumulation of both non-enzymatic and enzymatic antioxidants (Al-Rashed et al. 2016; Haghjou et al. 2009; Haghjou et al. 2006; Mohsenpour et al. 2012; Orefice et al. 2016; Sanchez-Saavedra and Voltolina, 2006; Schulze et al. 2016; Shu et al. 2012; Yilancioglu et al. 2014).

Most research on antioxidant enzyme activity in *Dunaliella* focuses on only one stressor with a few studies assessing the combined effect of two. There is a need for further research into this area to develop a deeper understanding of the ‘tuning parameters’ for enhanced enzymatic activity. Studies suggest that the combined effect of tuning several culture conditions is more effective for producing antioxidant responses in *Dunaliella* compared to exposure to a single stressor (Juneja et al. 2013; Al-Rashed et al. 2016; Lv et al. 2016). For instance, *D. salina* cells grown under UV-irradiation together with nitrogen limitation (El-Baky et al. 2004) or cells grown at low (13 °C) temperature combined with light irradiation (100 µmol photons m^−2^ s^−1^) (Haghjou et al. 2009) showed a higher enzymatic antioxidant activity compared to cells grown in controlled culture conditions. In addition, variation of light irradiation, extremely high or low temperature, high salinity, nutrient concentration (excess or starvation), UV irradiation or combination of culture conditions (low temperature and high light, UV irradiation and nitrogen starvation, high salinity with nitrogen starvation) have all been shown to enhance levels of non-enzymatic antioxidants (carotenoids, ascorbate, glutathione, tocopherol, phenolic) in *Dunaliella* (Gallego-Cartagena et al. 2019; Gómez and González 2005; Nguyen et al. 2016; Singh et al. 2016; Al-Rashed et al. 2016; Haghjou et al. 2009). Most research has focused on these non-enzymatic antioxidants rather than the enzymatic antioxidants production.

To use *Dunaliella* for antioxidant enzyme production, the culture conditions must be tuned to optimise the production of these high-value compounds. In this study, *D. tertiolecta* cells were exposed to three levels of each factor (light irradiation, temperature, nitrogen levels), and the combined effect on antioxidant productions was assessed. Enzymatic antioxidant (CAT, SOD, and peroxidase (POD)) activities and non-enzymatic antioxidant (phenolic, ascorbate, and glutathione) content in cell pellets were determined. To the best of our knowledge, this is the first report that describes the combined effect of light, temperature, and nitrogen level in enhancing enzymatic and non-enzymatic antioxidants production in *D. tertiolecta*.

## Materials and Methods

### Microalgae Species and Reagents

*D. tertiolecta* (CCAP 19/30) was obtained from the culture collection of Algae and Protozoa (CCAP, Scotland, UK). Modified Lowry assay kit (Lowry reagent and 2 N Folin-Ciocalteu reagent) was obtained from Thermo Scientific (UK), and all other reagents and enzymes were purchased from Sigma-Aldrich (UK).

### Factorial Design Experiment

A factorial experiment was designed to evaluate the effect of culture conditions on antioxidant responses in *D. tertiolecta*. Three light levels (15, 145, 550 µmol photons m^−2^ s^−1^), three temperatures (10, 20, 30 °C), and three different nitrogen levels (0.05, 0.5, 5 mM KNO_3_) were chosen as cultivation factors. Therefore, a total of 3^3^ = 27 experiments with different combinations of each factor were conducted (Table 1). All experiments were carried out in biological triplicate (*n* = 3). Cells (10 mL) for each flask were harvested on the 10th day after initiating the stress, and cell pellets were stored in labelled tubes at − 20 °C until further analysis. Initially, *D. tertiolecta* (1 × 10^5^ cells mL^−1^) were inoculated into modified Johnson medium (J/I) (pH 7.5) containing NaCl (87.66 g L^−1^) (Xu et al. (2016), and grown in glass flasks placed inside a static incubator at 20 °C ± 1 under LED (light-emitting diode) light (mixture of cool and warm white) irradiation (145 µmol photons m^−2^ s^−1^) with a light/dark cycle (12:12 h). Culturing flasks were shaken manually once a day during cell growth. Cells (1 × 10^6^ cells mL^−1^) at exponential growth phase were harvested by centrifugation (2000 × *g*, 10 min, 4 °C). Pellets were reconstituted into conical flasks (200 mL, 1 × 10^6^ cells mL^−1^) and were placed in a static incubator and exposed to the desired levels of continuous LED light irradiation, temperatures, and nitrogen levels as described in Table 1.

**Table 1:** Factorial design of light, temperature, and nitrogen levels

Experiment	Light (µmol photons m^−2^ s^−1^)	Temperature (°C)	Nitrogen concentration (mM KNO_3_)
1	15	10	0.05
2	15	10	0.5
3	15	10	5
4	15	20	0.05
5	15	20	0.5
6	15	20	5
7	15	30	0.05
8	15	30	0.5
9	15	30	5
10	145	10	0.05
11	145	10	0.5
12	145	10	5
13	145	20	0.05
14	145	20	0.5
15	145	20	5
16	145	30	0.05
17	145	30	0.5
18	145	30	5
19	550	10	0.05
20	550	10	0.5
21	550	10	5
22	550	20	0.05
23	550	20	0.5
24	550	20	5
25	550	30	0.05
26	550	30	0.5
27	550	30	5

### Cell Density

The cell number in cultures was counted using the Neubauer haemocytometer (Weber, UK). A cell suspension (0.015 mL) was mixed with a formalin solution (2%), and the total number of cells was determined.

### Pigment Content

Fresh cells (1 mL culture) were harvested by centrifugation (4000 × *g*, 4 °C, 10 min). The pellet was homogenised in extracting solution (80%v/v acetone) using a vortex mixer. The coloured supernatant was separated by centrifugation (14,000 × *g*, 4 °C, 10 min) and used for pigment analysis. The absorbance was measured against a blank of 80% acetone solution at 480, 647, and 663 nm using a spectrophotometer (Jenway 6305, UK). Chlorophyll a (Chl a), chlorophyll b (Chl b), and carotenoid contents in the acetone extract (µg mL^−1^) were calculated using the following relationship (Eqs. 1–4) (Takache et al. 2015; Xu et al. 2016).

1 $$\text{Total carotenoid}\left(\mu \text{g mL}-1\right)=4.0(\mathrm{Abs}_{480})$$

2 $$\begin{aligned}\text{Chlorophyll a} \left(\text{Chl a}\right)\left(\mu \text{g mL}-1\right)= &\left(12.25 \times \text{Abs}_{664}\right)\\&- (2.55 \times \text{Abs}_{647})\end{aligned}$$

3 $$\begin{aligned}\text{Chlorophyll b} \left(\text{Chl b}\right)\left(\mu \text{g mL}-1\right)=& \left(20.31 \times \text{Abs}_{647}\right)\\&- (4.91 \times \text{Abs}_{664})\end{aligned}$$

4 $$\begin{aligned}\text{Total chlorophyll}\left(\mu \text{g} \ \text{mL}^{-1}\right) =& \left(\text{ChL a}\right)\left(\mu \text{g} \ \text{mL}^{-1}\right)\\&+\left(\text{ChL b}\right)\left(\mu \text{g} \ \text{mL}^{-1}\right)\end{aligned}$$

where Abs_480_nm, Abs_647_nm, and Abs_664_nm are the absorbance of the acetone extract measured at 480, 647, and 664 nm, respectively.

### Phenolic Content

Fresh cells (10 mL culture) were harvested by centrifugation (1400 × *g*, 4 °C, 15 min), and the pellet was homogenised in deionised water (dH_2_O) (1 mL). After mixing (1 min, vortexed), the suspension was transferred to a sterile Eppendorf (1.5 mL) tube and incubated (30 min, sonication) following the protocol described by Falleh et al. (2012) and Machu et al. (2015) with some modifications. After centrifugation (14,000 × *g*, 4 °C, 15 min), the supernatant was kept on ice (10 min) and used for determination of total (water-soluble) phenolic content. Total phenolic content in the extract was determined as described by Kuda et al. (2005) using a standard calibration curve of gallic acid (0.1–8 µg mL^−1^). A cell extract (0.2 mL) was transferred to a clean plastic cuvette (*l* = 0.1 cm) containing Folin–Ciocalteu (FC) reagent (0.4 mL, 10% v/v) and incubated (3 min, room temperature). After adding Na_2_CO_3_ (10% w/v, 0.8 mL), the reaction mixture was incubated (1 h, room temperature) and the absorbance was taken at 750 nm (Jenway 6305, UK) against a blank sample containing dH_2_O (0.2 mL) instead of cells extract in the reaction mixture. The total phenolic compound in the crude extract was expressed as gallic acid equivalent (GAE).

### Protein Content

Fresh cells (10 mL culture) were harvested by centrifugation (1400 × g, 4 °C, 15 min), and the pellet was homogenised in NaOH (5 mL, 0.1 mM). After incubation (continuous agitation, 250 rpm, 45 min, 40 °C), the supernatant was separated by centrifugation (21,000 × *g*, 4 °C, 30 min) and collected as the first extract. A second extraction was carried out for the residue, and the second supernatant was mixed with the first extract and used for protein assay. Total protein content in the crude extract was determined by the method of Lowry assay (Martina and Vojtech 2015) using ovalbumin as the protein standard.

### H_2_O_2_ Content

Fresh cells (10 mL culture) were harvested by centrifugation (1400 × *g*, 4 °C, 15 min), and the pellet was homogenised in a 1.0 mL reaction mixture (trichloroacetic acid (TCA), 0.25% w/v; potassium iodide (KI), 25 mM; phosphate buffer, 2.5 mM, pH 7.0). After mixing (3 min, vortex), the homogenate was transferred to a sterile Eppendorf tube (1.5 mL) and incubated (20 min) on an ice bath in the dark. After centrifugation (12,000 × *g*, 4 °C, 15 min), the supernatant was incubated (20 min, room temperature) and the absorbance was recorded at 350 nm against blank prepared by adding dH_2_O (0.5 mL) instead of KI into the reaction mixture (Junglee et al. 2014). A calibration curve for quantifying hydrogen peroxide (H_2_O_2_) content was prepared using standard solutions of H_2_O_2_ (0.06–0.310 nM) and estimated as above.

### Lipid Peroxide (MDA) Content

Fresh cells (10 mL culture) were harvested by centrifugation (1400xg, 4 °C, 15 min) and the pellet was homogenised in 1.4 mL reaction mixture (Thiobarbituric acid (TBA), 0.3%w/v; TCA, 3.9%w/v). After mixing (3 min, vortex), the homogenate was transferred to a sterile Eppendorf (1.5 mL) tube and heated (30 min, 95 °C). The mixture was incubated (10 min) on ice. After centrifugation (12,000 × *g*, 4 °C, 10 min), the absorbance of the supernatant was recorded (532 and 600 nm) using a spectrophotometer (Jenway 6305, UK). A blank sample containing 1.4 mL reaction mixture (TBA, 0.3% w/v; TCA, 3.9% w/v) was also prepared. In this assay, total lipid peroxide content was quantified as malondialdehyde (MDA) levels (nmoL 10^−6^ cells) and calculated using the following formula (Eq. 5) (Hodges et al. 1999; Shah et al. 2001).

5 $$\text{MDA equivalents (nmoL }\text{mL}{-1 }) = \frac{(\text{A}{532}-\text{A}\text{600})\times {10}{-6}}{{ (155},{000)}}$$

where 532 nm represented the maximum absorbance of the TBA-MDA complex; 600 nm represented the correction for non-specific turbidity; 155,000 (M^−1^ cm^−1^) = the molar extinction coefficient of MDA.

### Ascorbate and Dehydroascorbate Content

Fresh cells (10 mL culture) were harvested by centrifugation (1400 × *g*, 4 °C, 15 min), and the pellet was homogenised in TCA (0.5 mL, 10% w/v) and transferred into a sterile and clean Eppendorf tube (1.5 mL). The tube was shaken (5 min) vigorously using a vortex mixer and incubated (10 min) on ice. After centrifugation (4000 × *g*, 4 °C, 10 min), the supernatant was collected as crude extract and kept on ice for spectrophotometric determination ascorbate (ASc) and dehydroascorbate (DASc) content. An aliquot of the cell extract (0.1 mL) was transferred to a clean plastic cuvette (*l* = 1 cm) containing 0.9 mL assay mixture (phosphate buffer (0.25 mL, 100 mM, pH 7.00), ethylenediaminetetraacetic acid (EDTA) (100 mL, 10 mM), and dH_2_O (0.5 mL)). After adding FC reagent (0.2 mL, 10% v/v), the reaction mixture was shaken vigorously (5 min, vortexing, room temperature) and the absorbance was measured (760 nm) against a blank sample containing TCA (0.1 mL, 10%) instead of cells extract (Jagota and Dani 1982). A calibration curve of a standard solution of ascorbic acid (in dH_2_O) (0.5–5 µg) was used for quantifying ascorbate content.

An aliquot of cell extract (0.1 mL) was transferred to a clean plastic cuvette (*l* = 1 cm) containing 0.85 mL assay mixture (phosphate buffer (0.25 mL, 100 mM, pH 7.00), EDTA (100 mL, 10 mM), dH_2_O (0.400 mL), and DTT (30 mM, 0.05 mL)) and incubated (10 min, room temperature) to facilitate the reduction of DASc to ASc. After adding N-ethylmaleimide (NEM) (0.5% w/v, 0.05 mL), the reaction mixture was incubated (5 min, room temperature) and FC reagent (0.2 mL, 10% v/v) was added. The reaction mixture was shaken vigorously (5 min, vortexing, room temperature), and the absorbance was measured (760 nm) against a blank sample containing TCA (0.1 mL, 10%) instead of cells extract (Jagota and Dani 1982). Total reduced ascorbate (TASc) was calculated from the calibration curve. The DASc content was estimated by subtraction of the amount of reduced ascorbate (ASc) from the TASc content.

### Total Glutathione Content and Reduced Flutathione Content

Fresh cells (10 mL) were harvested by centrifugation (1400 × g, 4 °C, 15 min), and the pellet homogenised in 0.5 mL extraction buffer (50 mM sulfosalicylic acid, 1 mM EDTA, and 8.5 mM ascorbic acid) followed by cell disruption by vortexing in the presence of silica beads and sonication (2 min). After centrifugation (14,000 × *g*, 4 °C, 15 min), the supernatant was collected in a sterile Eppendorf tube (1.5 mL) and kept on ice for determination of glutathione content.

An aliquot of cell extract (0.2 mL) was transferred to a clean plastic cuvette (*l* = 1 cm) containing 0.9 mL assay mixture (5,5-dithiobis-2-nitrobenzoic acid (DTNB), 8.6 mM, glutathione reductase (GR) 2.73U in phosphate buffer (100 mM, pH 7.00)). Phosphate buffer (0.1 mL, 100 mM, pH 7.00) was added to the reaction mixture and incubated (30 min, room temperature). After adding NADPH solution (0.75 mL, 0.827 mM) and mixing (gentle inversion), the cuvette was placed inside the spectrophotometer (Jenway 6305, UK). The change in optical density (ΔOD) due to the production of 5-thio-2-nitrobenzoic acid (TNB) was recorded (0 min, 5 min) at 412 nm against a blank sample containing extraction buffer instead of cell extract. A calibration curve for quantifying total glutathione (TGSH) was prepared using standard reduced glutathione (0.312–25 nmol). The TGSH content in cell extract was calculated using Eq. 6 (Salbitani et al. 2017).

Reduced glutathione (GSH) level was estimated using the same procedure as TGSH but excluding the GR and NADPH in the reaction mixture. The reaction mixture (1.275 mL) contained DTNB (0.9 mL, 8.6 mM) in phosphate buffer (100 mM, pH 7.00), phosphate buffer (0.175 mL, 100 mM, pH 7.00), and cell extract (0.2 mL). DTNB is converted into TNB in this assay due to the only participation of GSH. After incubation (5 min, room temperature) and mixing gently (by inversion), the absorbance of the reaction mixture recorded spectrophotometrically (412 nm) against a blank sample containing extraction buffer instead of cells extract. GSH content was estimated using Eq. 6.

6 $$\begin{aligned}&\text{Total glutathione content}\left[\text{nmol}\left(10^{-6}\;\text{cells}\right)\right]\\&=\frac{\left(\Delta{\text{OD}}_\text{s}\right)\times\text{total volume}\left(\text{mL}\right)}{\left(\Delta{\text{OD}}_\text{std}\right)\times\text{Sample volume }\left(\text{mL}\right)\times\text{length }\left(\text{cm}\right)}\end{aligned}$$

where ΔOD_S_ = optical density changes due to GSH in sample; ΔOD_Std_ = optical density changes due to standard GSH solution.

### Determination of Antioxidant Enzyme Activity

#### Preparation of Crude Extract

Crude extract for enzymes assay (CAT, POD, and SOD) were prepared from *D. tertiolecta* fresh cell pellet (culture (10 mL) harvested by centrifugation (3000 × *g*, 15 min, 4 °C) using a modified method described by Tian and Yu (2009). A cell pellet was dissolved in an extraction buffer (1 mL, 50 mM potassium phosphate buffer (0.1 mM EDTA, 0.1% Triton X-100, 1% polyvinylpyrrolidone (PVP), and pH 7.5) and transferred into a sterilized Eppendorf tube (1.5 mL). The homogenate was vortexed (5 min) in the presence of silica beads (0.2 g), and the supernatant was separated after centrifugation (13,000 × *g*, 30 min, 4 °C). The supernatant was used in the subsequent analysis for the antioxidant enzyme activity. Total protein content in crude extract was measured using Lowry assay with ovalbumin as protein standard.

#### CAT Activity

Extract (0.05 mL) were transferred to a quartz cuvette (*l* = 0.1 cm) containing ice-cold phosphate buffer (1 mL, 50 mM, pH 7.00) (Zhang et al. 2015). The reaction mixture was equilibrated (25 °C, 5 min) and H_2_O_2_ (0.05 mL, 858 mM) was added (final concentrations H_2_O_2_ 0.045 mM and phosphate buffer 39 mM). After mixing (gentle inversion), decrease in optical density (ΔOD) was recorded (every 10 s, 5 min, 240 nm) against a blank sample containing dH_2_O in the reaction mixture instead of extract. The specific CAT activity was calculated as units per milligram of protein (Tian and Yu 2009), Eq. 7.

7 $$\begin{aligned}&\text{CAT activity} (\text{U mg}{-1}\text{ protein)} \,\\& = \frac{\left(\text{OD min}{-1}\right) \, \times \text{ total volume }\left({\text{mL}}\right) \, \times \text{ df}}{{0.0436 }\times \text{ sample volume }\left({\text{mL}}\right) \, \times \text{ protein content} ({\text{mg mL}}{-1}) \times \text{ length }\left({\text{cm}}\right)}\end{aligned}$$

where 0.0436 = millimolar extinction coefficient of H_2_O_2_ at 240 nm, df = dilution factor.

#### POD Activity Assay

ABTS (2,2′-azino bis (3 ethylbenzthiazoline-6-sulfonic acid) solution (0.964 mL, 9.1 mM) in phosphate buffer (100 mM, pH 5.00) and extract (0.01 mL) was transferred to a quartz cuvette (*l* = 0.1 cm) and placed inside the spectrophotometer. After equilibration (25 °C, 5 min) of the reaction mixture, H_2_O_2_ (0.036 mL, 88.5 mM) was added, and the change in optical density (ΔOD) was recorded (every 10 s, 3 min, 420 nm) against a blank sample containing dH_2_O in the reaction mixture instead of extract. The specific POD activity was calculated as units per mg of protein, Eq. 8.

8 $$\begin{aligned}&\text{POD activity} (\text{U mg}{-1}\text{ protein}) \,\\& = \frac{(\text{OD min}{-1}) \times \text{ total volume }\left({\text{mL}}\right) \, \times \text{ df}}{{36.8 }\times \text{ sample volume }\left({\text{mL}}\right) \, \times \text{ protein content} ({\text{mg mL}}{-1}) \times \text{ length }\left({\text{cm}}\right)}\end{aligned}$$

where 36.8 = millimolar extinction coefficient of ABTS, df = dilution factor.

#### SOD Activity Assay

Phosphate buffer (0.667 mL, 75 mM, pH 7.00), dH_2_O (0.06 mL), xanthine (0.1 mL, 150 mM), and NBT (0.1 mL, 1.5 mM) was transferred into a quartz cuvette (d = 0.1 cm). After equilibration (25 °C, 5 min) of the reaction mixture, xanthine (0.023 mL, 0.4 U mL^−1^) oxidase was added to it, and the optical density (ΔOD) was recorded (per second, 3 min, 560 nm) against a blank sample containing dH_2_O in reaction mixture instead of xanthine oxidase. After 3 min, the extract (0.05 mL) was added into the reaction mixture, and the decrease in optical density (ΔOD) was recorded up to 7 min. The specific SOD activity was calculated as units per mg of protein using the following formula, Eqs. 9–10.

9 $$\begin{aligned}&\text{Percent inhibition} (\%) \,\\&=\frac{(\Delta\text{ODs min}-1\,-\,\Delta\text{ODO min}-1)\times100}{(\Delta\text{ODs min}{-1})}\end{aligned}$$

Increase in absorbance (uninhibited) per min (ΔOD_s_ min^−1^) at 560 nm.

Inhibition of absorbance per min (ΔOD_o_ min^−1^) by the sample at 560 nm.

10 $$\begin{aligned}&\text{SOD activity} (\text{U mg}-{1}\text{ protein}) \text{in extract } \,\\& = \frac{\text{Percent inhibition }\times \text{ df}}{{50\% }\times \text{ sample volume} (\text{mL}) \, \times \text{ U mg}{-1}\text{ protein}}\end{aligned}$$

### Statistical Analysis

The individual and the interactive effect of light, temperature, and nitrogen concentration on all accumulated biomolecules including antioxidant enzyme activity were evaluated using 3^3^ factorial ANOVAs with light levels (15, 145, 550 µmol photons m^−2^ s^−1^), temperature (10, 20, 30 °C), and nitrogen level (0.05, 0.5, 5 mM KNO_3_) as experimental factors (SPSS software (IBM, USA). A *t*-test (non-equal variance) was performed when comparing two means. R software (The comprehensive R archive network) was used to confirm normality. Tukey’s HSD was carried out for pairwise comparison of factors. Graphical data are represented as means and standard error. To predict the best combination of cultivation factors to obtain maximum individual antioxidant content in *D. tertiolecta*, Design-Expert experiments (DoE) (full factorial) was conducted using Minitab Statistical Software (Minitab®, Pennsylvania State University).

## Results

The combined effect of light irradiation (chemical energy source), temperature (intracellular enzymatic reactions and biochemical composition), and nitrogen (growth and cellular metabolism) were used to induce oxidative stress to stimulate enzymatic and non-enzymatic antioxidant contents in *D. tertiolecta.* To assess the overall intracellular antioxidant response, the accumulation of chlorophyll content, cell density, total protein, H_2_O_2_ and MDA content, including enzymatic (CAT, SOD, and POD and non-enzymatic antioxidant (carotenoids, phenolic, ascorbate, and glutathione) levels, were determined.

### Cell Density

**Fig. 1:**
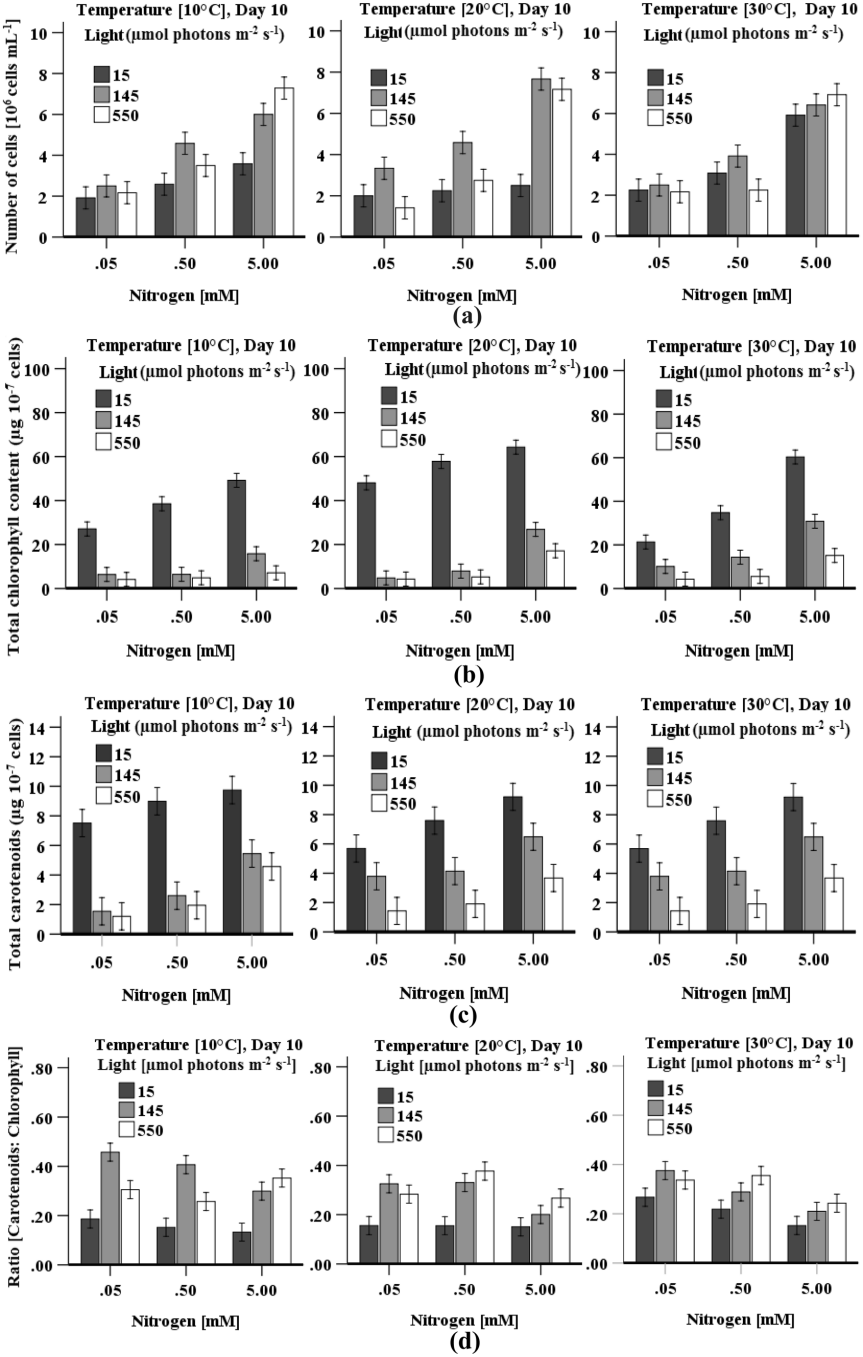
Effect of light, temperature, and nitrogen concentration on **a** cell density, **b** chlorophyll, **c** carotenoid content, and **d** ratio of chlorophyll to carotenoids (Car:Chl) in *D. tertiolecta* cultures. Data is presented as mean ± SE (*n* = 3)

Cell density (biomass production) was directly influenced by light irradiation and nitrogen concentration during culturing of *D. tertiolecta* cells (Fig. 1a). Cell density consistently increased with nitrogen concentration from 0.05 to 5 mM KNO_3_ under any combination of light and temperature except from cells grown with 0.5 M KNO_3_ at 30 °C under 550 µmol photons m^−2^ s^−1^. Cells grown with high nitrogen (5 mM KNO_3_) at light irradiation of 145 or 550 µmol photons m^−2^ s^−1^ showed significantly (*p* < 0.01) higher cell density compared to cells exposed to low light irradiation (15 µmol photons m^−2^ s^−1^) at all three temperatures (Fig. 1a). The highest cell density (~ 8 × 10^6^ cells mL^−1^) was observed in cells grown at 20 °C under 145 µmol photons m^−2^ s^−1^ with high nitrogen content (Fig. 1a); however, no significant (*p* > 0.05) difference in cell densities were observed in cells grown at 10 °C under 550 µmol photons m^−2^ s^−1^ with a high nitrogen content, and in cultures grown at 30 °C under 145 or 550 µmol photons m^−2^ s^−1^ with a high nitrogen content. A three-way ANOVA illustrates that all individual factors except for temperature (ANOVA, *p* = 0.11, df = 2, 54) and all interactive factors had a highly significant influence on cell density (*p* < 0.0001).

### Chlorophyll and Total Carotenoid Content

The accumulation of chlorophyll and total carotenoid content consistently increased with increasing nitrogen concentration and consistently decreased with increasing light irradiation (Fig. 1b, c). Chlorophyll production was also enhanced when cells were grown at 20 °C under any combination of light and nitrogen compared to cultivation at either 10 °C or 30 °C, whereas carotenoid production was highest under all condition when cells where cultivated at 10 °C. The highest chlorophyll content (65 ± 5.14 µg 10^−7^ cells) was observed in cells grown at 20 °C under 15 µmol photons m^−2^ s^−1^ with high nitrogen levels (Fig. 1b), whereas the highest carotenoid content (9.75 ± 1.65 µg 10^−7^ cells) was achieved at 10 °C under the same light and temperature (Fig. 1c). A three-way ANOVA for both chlorophyll and carotenoid content indicates that all individual or combing factors are statistically significant, except for the interaction of light × temperature × nitrogen (ANOVA, *p* = 0.74, df = 4, 54) and light × nitrogen (ANOVA, *p* = 0.10, df = 2, 54) which did not have a significant impact on carotenoid content. The Car:Chl ratio increased with a decrease in nitrogen levels and an increase in light irradiation (Fig. 1d). A highest Car:Chl ratio (Fig. 1d) was found in cells grown with low nitrogen (< 0.5 mM) under 145 µmol photons m^−2^ s^−1^. Conversely, the lowest Car:Chl ratio (0.133) was observed when cells were grown with high nitrogen under low light irradiation, which represents high chlorophyll accumulation.

### Protein Content

**Fig. 2:**
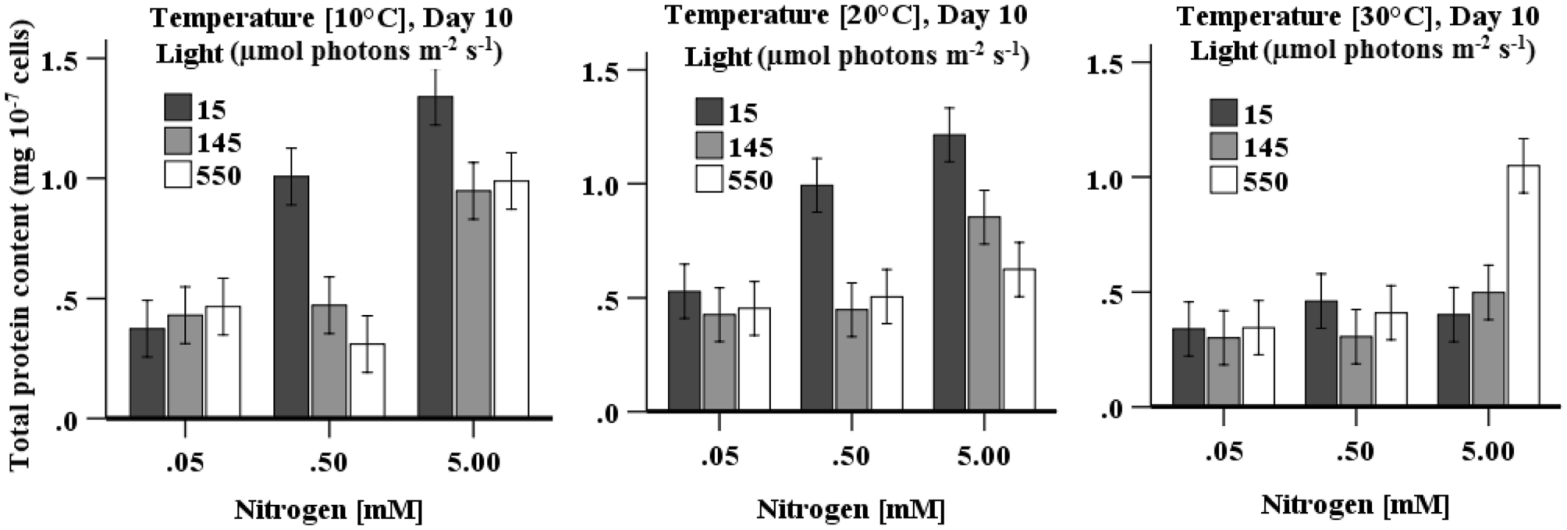
Effect of light, temperature, and nitrogen concentration on total protein in *D. tertiolecta* cultures. Data is presented as mean ± SE (*n* = 3)

Protein content increased as the nitrogen concentration in the medium increased regardless of the changes to other cultivation factors (Fig. 2) except for cultivations with 0.5 mM KNO_3_ at 10 °C under 550 µmol photons m^−2^ s^−1^, cultivation with 0.05–0.5 mM KNO_3_ under 15 or 145 µmol photons m^−2^ s^−1^ at 30 °C. Protein content was predominantly highest when cells were cultured at either 10 or 20 °C with a nitrogen concentration of 5 mM KNO_3_. The highest protein content (1.41 ± 0.27 mg 10^−7^ cells) was obtained in cells that were grown at 10 °C under low light irradiation (15 µmol photons m^−2^ s^−1^) and with high nitrogen content (5.0 mM) (Fig. 2). A three-way ANOVA indicates that all three factors (individual or interactive) have a statistically significant (*p* < 0.05) impact on protein accumulation, which indicates in the importance in correctly tuning these parameters to enhance protein content. To obtain maximum total protein levels in *D. tertiolecta*, cells should be cultured in a high nitrogen-containing medium at low temperature and under low light irradiation.

### H_2_O_2_ and MDA Content

**Fig. 3:**
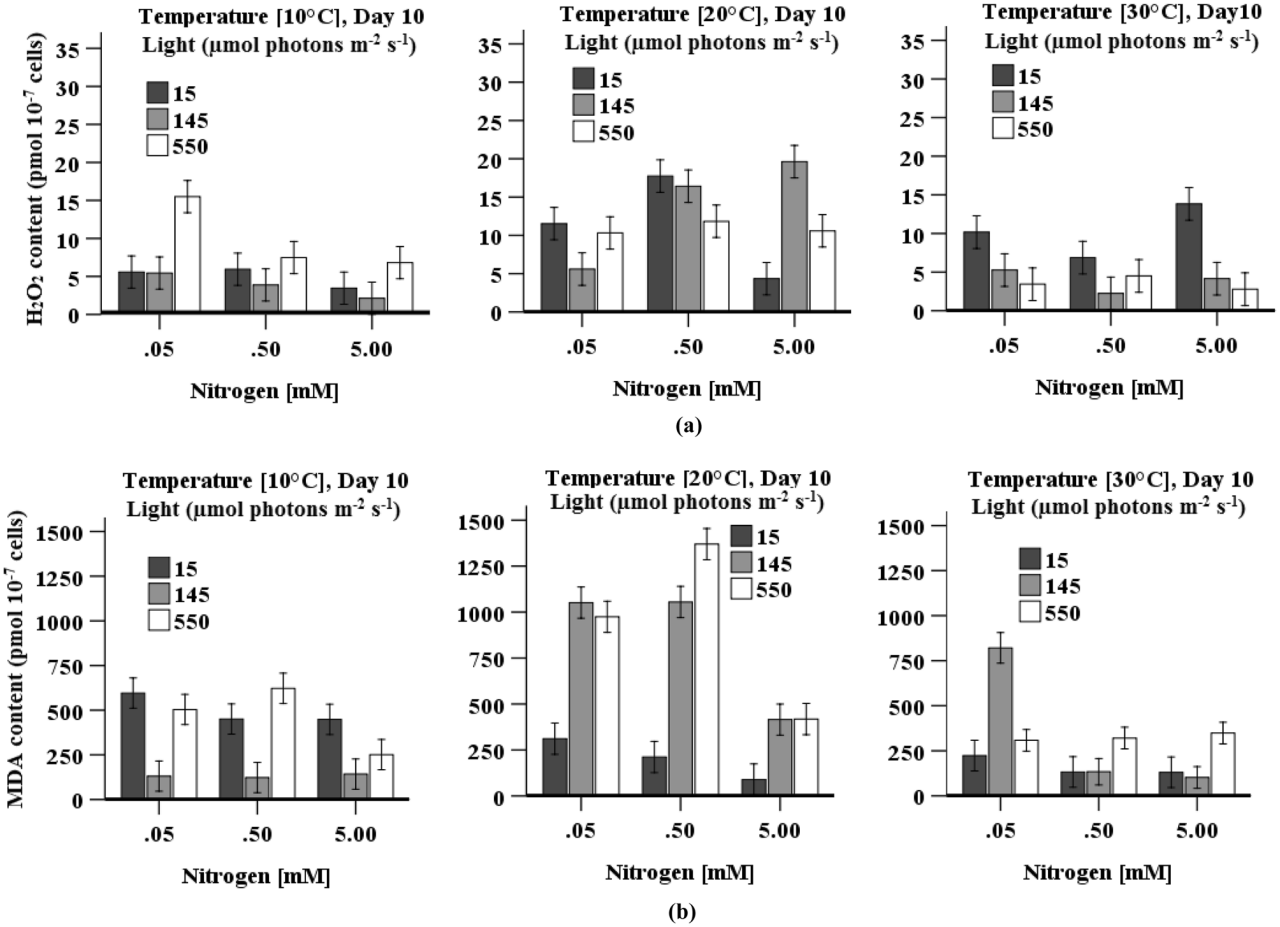
Effect of light, temperature, and nitrogen concentration on accumulation of **a** H_2_O_2_ and **b** MDA content in *D. tertiolecta* cultures. Data is presented as mean ± SE (*n* = 3)

Maximum H_2_O_2_ content (19.63 ± 3.63 pmol 10^−7^ cells) was found when cells were grown with a high nitrogen concentration under medium light irradiation (145 µmol photons m^−2^ s^−1^) and at 20 °C though not significant different from the H_2_O_2_ content (10.32 ± 1.12 pmol 10^−7^ cells) accumulated in cells cultivated at high light and low nitrogen concentrations (Fig. 3a). A three-way ANOVA indicates that all individual or interactive factors, except for nitrogen concentration, are statistically significant in accumulating H_2_O_2_ content in cells. The highest MDA content (1376 ± 300 pmol 10^−7^ cells) was found when cells were grown at 20 °C with 0.5 mM KNO_3_ under 550 µmol photons m^−2^ s^−1^. Though MDA content (1057 ± 277 pmol 10^−7^ cells) did not significantly change in cells when grown with 0.05 mM KNO_3_ under medium light irradiation relative to cells grown with nitrogen concentration (0.5 mM), these contents were significantly (*p* < 0.05) higher relative to cells grown with high nitrogen levels (5 mM KNO_3_) under similar light irradiation. The data indicate that depletion of nitrogen predominantly induces oxidative stress and subsequently increases the levels of MDA. A three-way ANOVA indicates that all three factors (individual or interactive) have a statistically significant impact on the accumulation of MDA content in *D. tertiolecta.* H_2_O_2_ content (15.23 ± 3.61 pmol 10^−7^ cells) was significantly (*p* < 0.05) higher at 10 °C when cells were cultured with low nitrogen levels under high light irradiation relative to cells grown under low or medium light irradiation with the same temperature and nitrogen levels. Conversely, a significantly (*p* < 0.05) higher MDA content at 10 °C was obtained in cells when cultured under both low or high light irradiation relative to cells grown under medium light irradiation at the same nitrogen and temperature.

### Non-enzymatic Antioxidants

#### Phenolic content

**Fig. 4:**
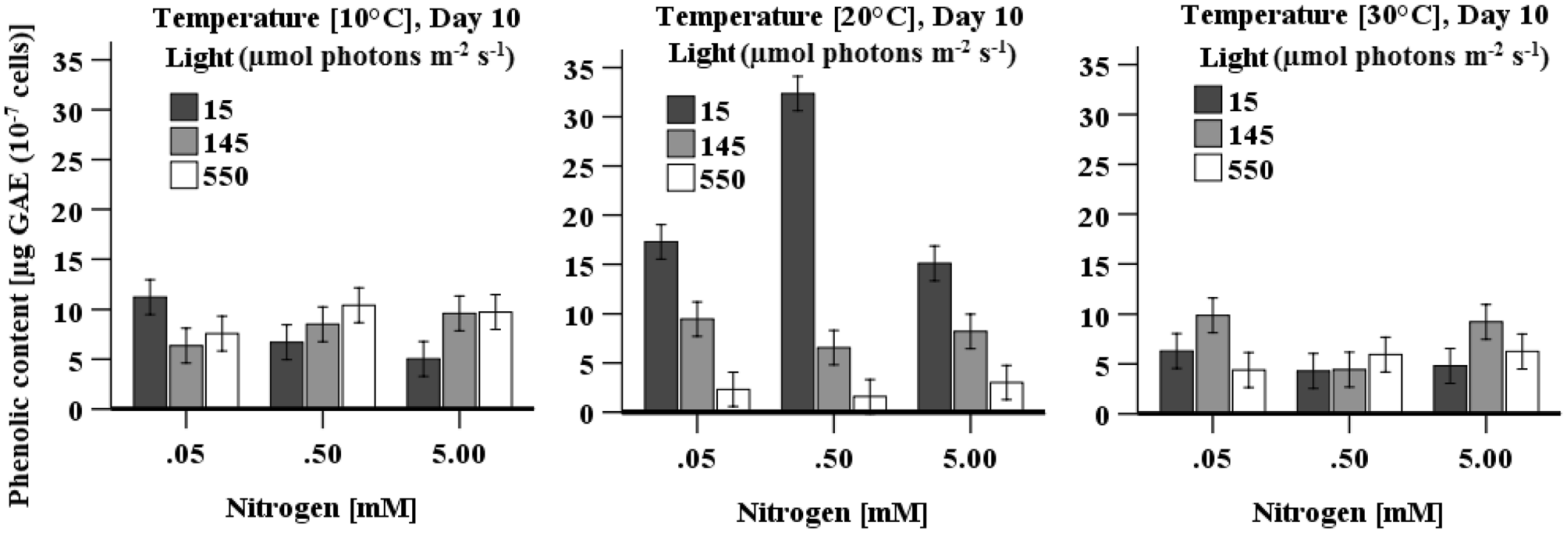
Effect of light, temperature, and nitrogen concentration on total phenolic contents in *D. tertiolecta* cultures. Data is presented as mean ± SE (*n* = 3)

The highest phenolic content (32 ± 2.6 µg GAE 10^−7^ cells) was achieved when cells were cultured at 20 °C under low light irradiation and with a nitrogen concentration of 0.5 mM KNO_3_ (Fig. 4). Unexpectedly, under 20 °C cultivation, accumulation of phenolic compounds decreased as light irradiation increased, and the lowest phenolic content was found when cells were grown under high light irradiation (550 µmol photons m^−2^ s^−1^) at all three nitrogen levels. This was opposite in cells cultivated at 10 °C and with > 0.5 mM nitrogen where a significantly (*p* < 0.05) higher phenolic content was observed in cells cultivated under high light irradiation levels relative to cells cultured under low light irradiation (Fig. 4). A three-way ANOVA indicates that all three factors (individuals and interactive) have a statistically significant (*p* < 0.05) impact on phenolic accumulation in cells.

#### Ascorbate and reduced ascorbate content

**Fig. 5:**
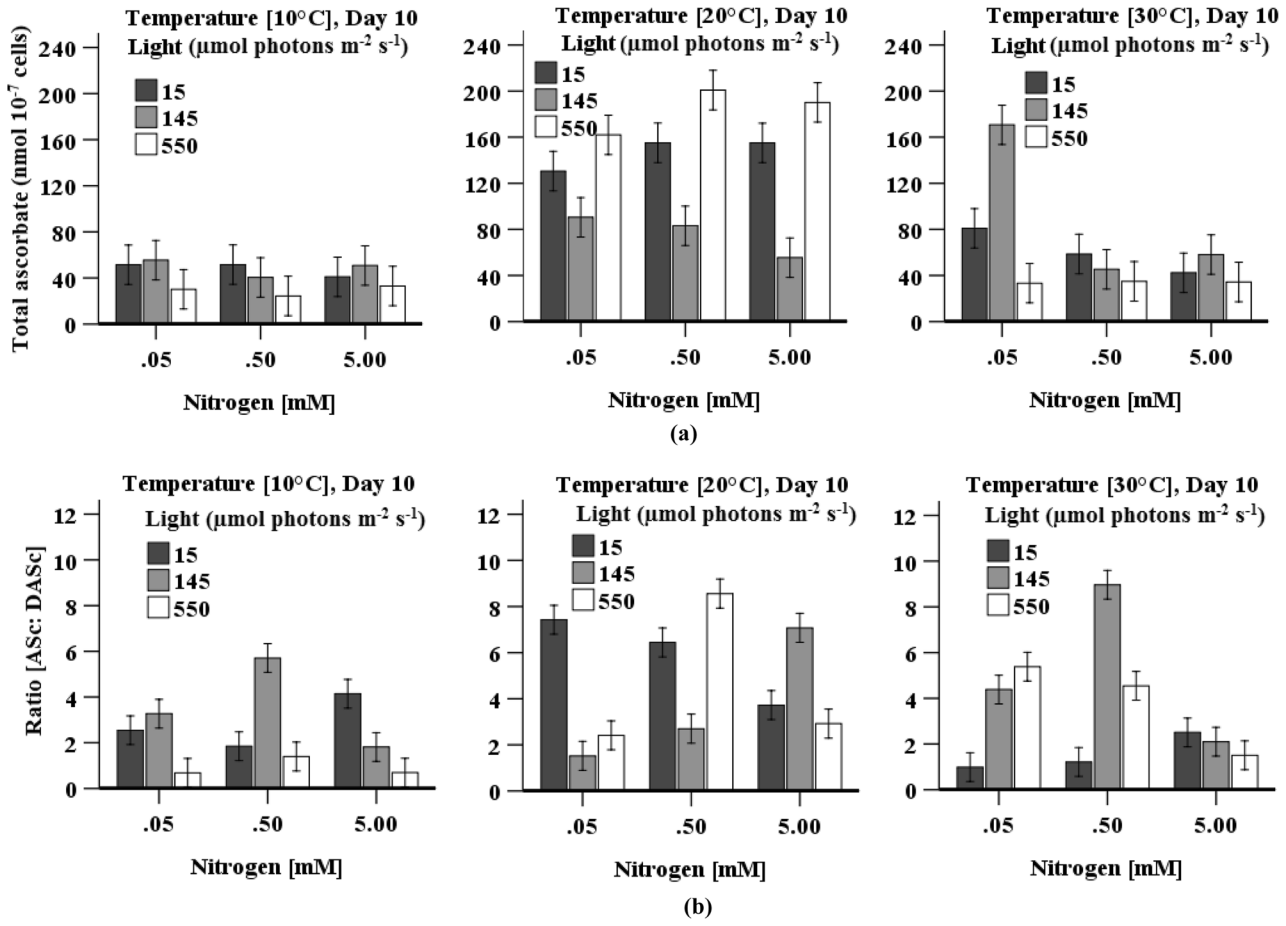
Effect of light, temperature, and nitrogen concentration on **a** total ascorbate content and **b** ratio of ASc:DASc in *D. tertiolecta* cultures. Data is presented as mean ± SE (*n* = 3)

Ascorbate pool (reversible inter-conversion of reduced ascorbate (ASc) to dehydroascorbate (DASc)) is involved in the intracellular physiological process (either as an antioxidant for direct detoxification of ROS or act as an electron donor for ascorbate peroxidase enzyme activity), and levels are influenced by manipulation of factors (light, temperature, and nitrogen concentration) during cultivation. The accumulation of total ascorbate content was significantly higher (*p* > 0.05) in cells when grown at 20 °C under all light or nitrogen concentrations compared to either 10 or 30 °C except for the cell growth at 30 °C with low nitrogen under medium light irradiation (Fig. 5a). In addition, content was significantly higher at 20 °C, under low or high light irradiation relative to medium light irradiation at the same nitrogen concentration, and maximum ascorbate content (200 ± 17 nmol 10^−7^ cells) was achieved in cells grown with 0.5 mM KNO_3_ under high light irradiation. Though variation in nitrogen concentration (0.5–5 mM KNO_3_) at 20 °C did not have a significant impact on total ascorbate content in cells, significantly (*p* > 0.05) lower ascorbate was found when cells were cultured with low nitrogen (0.05 mM KNO_3_) under low or high light irradiation. A significantly (*p* < 0.05) higher ratio of ASc:DASc was observed in cells grown at 20 °C under high light irradiation (Fig. 5b) with nitrogen levels of 0.5 mM KNO_3_ relative to cells cultured with low or high nitrogen under the same light irradiation (Fig. 5b). In contrast, compared to high nitrogen, significantly (*p* < 0.05) lower ratio of ASc:DASc was observed in cells grown with low nitrogen under light irradiation of 145 µmol photons m^−2^ s^−1^ at 20 °C, which indicates high DASc content in the cells.

Maximum total ascorbate (170 ± 32 nmol 10^−7^ cells) content was observed at 30 °C when cells were grown with low nitrogen under medium light irradiation (Fig. 5a). An increase in ASc:DASc ratio with decreasing nitrogen levels was found when cells were grown at 30 °C under > 145 µmol photons m^−2^ s^−1^, and this ratio was maximum when cells were cultured with medium nitrogen (0.5 mM KNO_3_) under medium light irradiation.

#### Total Glutathione and Reduced Glutathione Content

**Fig. 6:**
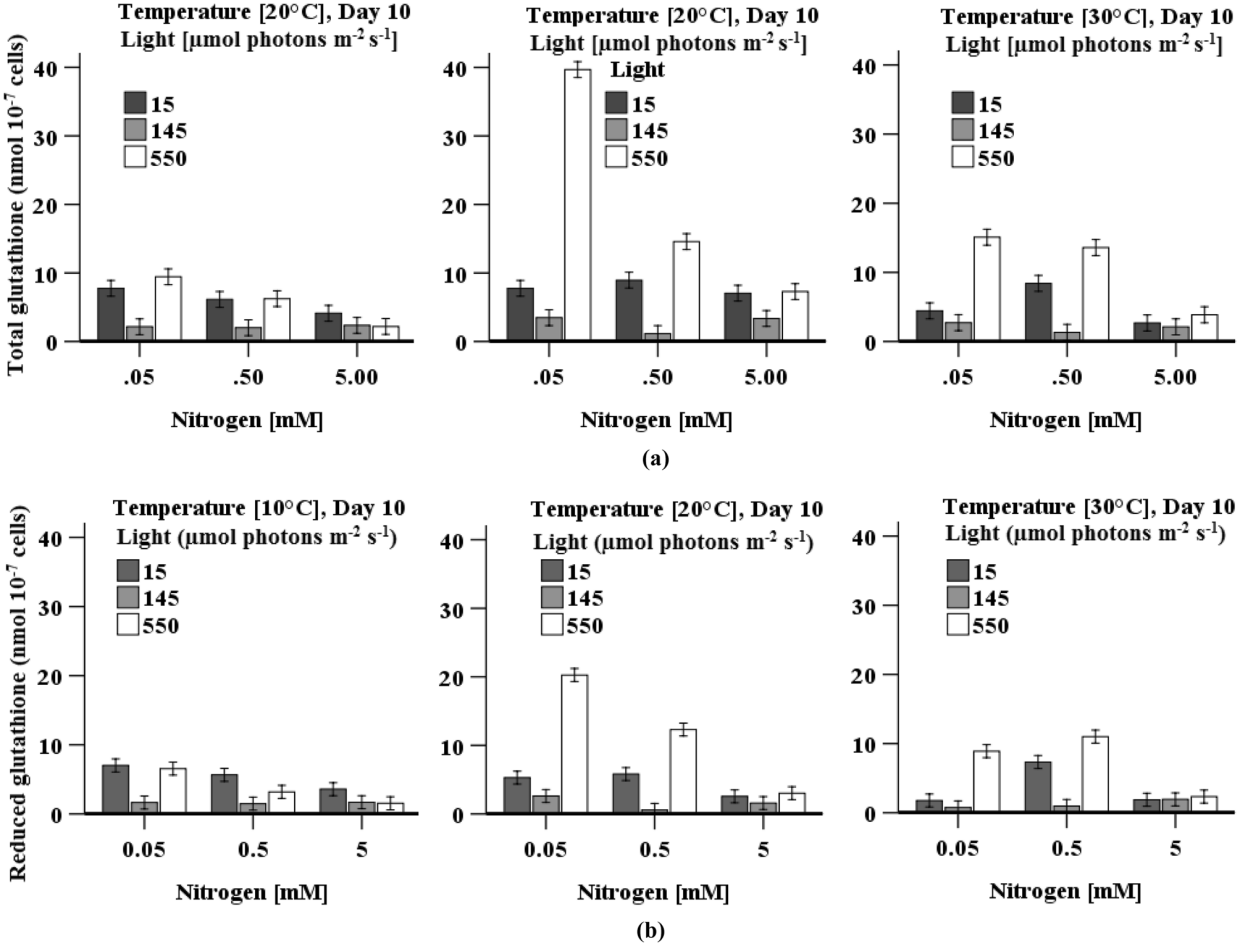
Effect of light, temperature, and nitrogen levels on **a** accumulation of total glutathione and **b** reduced glutathione content in *D. tertiolecta* cultures. Data is presented as mean ± SE (*n* = 3)

The accumulation of total glutathione (oxidised + reduced) and GSH content gradually increased with a decrease in nitrogen concentration under all three levels of light irradiation (Fig. 6a, b). A three-way ANOVA indicate that all individual or interactive factors are statistically significant in accumulating total glutathione content in cells. The concentration of these compounds was significantly (*p* < 0.05) higher in cells when grown with low nitrogen under low but especially high light irradiation relative to cells cultured under medium light (145 µmol photons m^−2^ s^−1^) with the same temperature. Maximum total glutathione (39 ± 2 nmol 10^−7^ cells) and reduced glutathione content (20 ± 2 nmol 10^−7^ cells) was found in cells grown at 20 °C with low nitrogen under high light irradiation. Nitrogen concentration did not have significant impact on the accumulation of total glutathione or reduced glutathione content in cells when grown under medium light irradiation at the three temperature (Fig. 6a, b).

### Antioxidant Enzyme Activity

#### CAT Activity

**Fig. 7:**
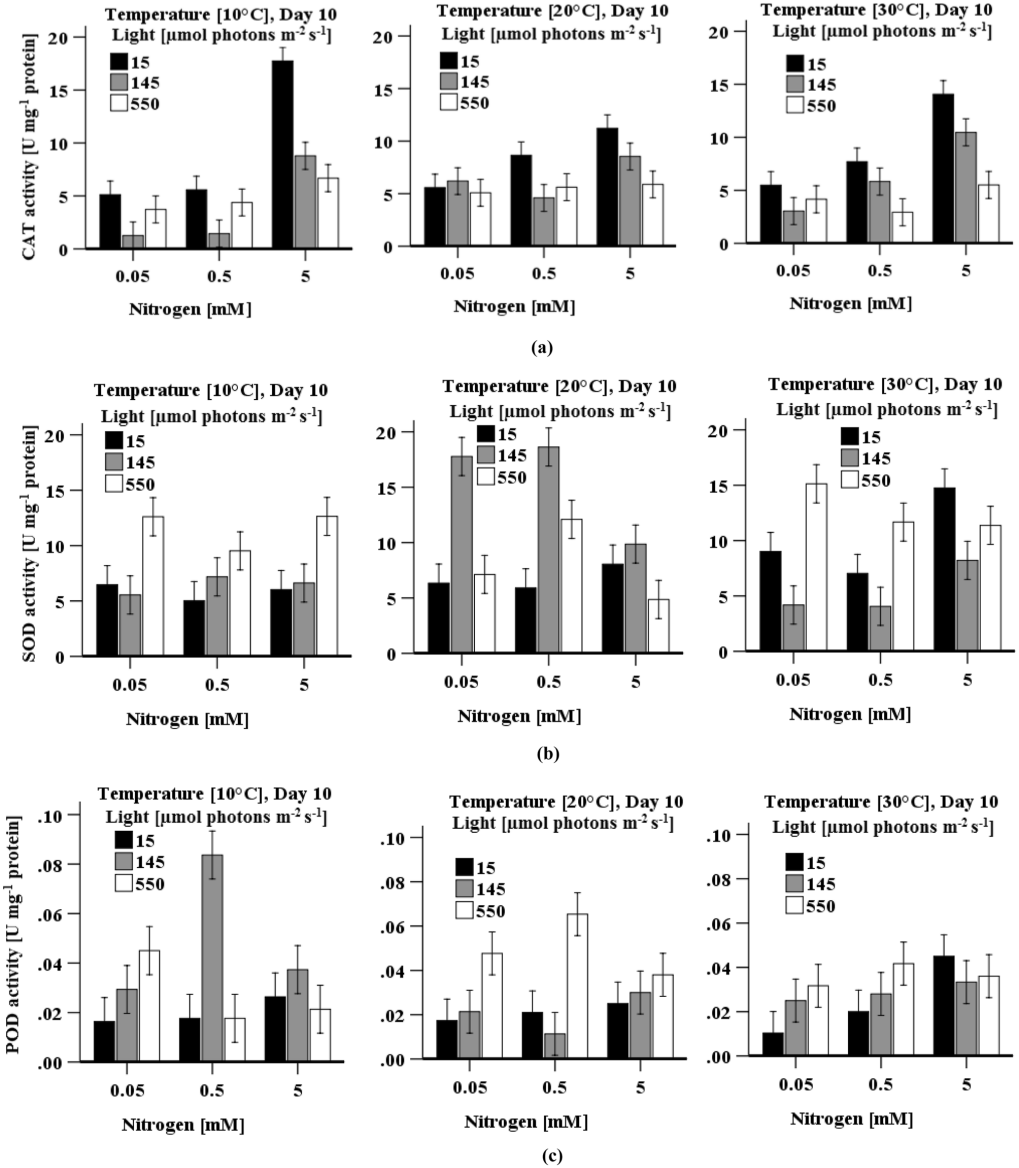
Effect of light, temperature, and nitrogen levels on **a** CAT activity, **b** SOD activity, and **c** POD activity in *D. tertiolecta* culture. Data is presented as mean ± SE (*n* = 3). Bars show standard error, predicted from ANOVA mode

CAT activity was significantly (*p* < 0.05) higher in cells when grown with high nitrogen under any levels of light and temperature relative to cells grown with low nitrogen under the same light and temperature. Though the variation of temperature did not have significant effect on CAT activity in cells grown with high nitrogen concentration under high light irradiation, activity was significantly (*p* < 0.05) higher when cells were cultured with high nitrogen at 10 °C under low light irradiation relative to cells grown at 30 °C under the same light and nitrogen levels. A three-way ANOVA illustrates that light intensity (*p* < 0.001) and nitrogen concentration (*p* < 0.001) have a significant effect on CAT activity in cultivated cells. However, the effect of temperature (*p* = 0.053) was not significant. The interactions of light × temperature, light × nitrogen, temperature × nitrogen, light × temperature × nitrogen all have a significant (*p* < 0.001) effect on CAT activity. CAT activity increased with a decrease in light irradiation at all three temperatures tested (Fig. 7a). Maximum CAT activity (17.75 ± 1.56 U mg^−1^ protein) was achieved when cells were grown with high nitrogen at 10 °C under low light irradiation (Fig. 7a).

#### SOD Activity

A three-way ANOVA indicates that light intensity and temperature (*p* < 0.001) significantly affect SOD activity in cells. However, nitrogen concentration is not statistically (*p* = 0.080) significant. The interactions of light × temperature, light × nitrogen, temperature × nitrogen, and light × temperature × nitrogen all have significant (*p* < 0.001) effect on SOD activity in cells cultivated for 10 days. Indeed, at 10 °C, increases in both light and nitrogen levels resulted in an increased SOD activity, although not at 145 µmol photons m^−2^ s^−1^, 0.05 mM KNO_3_ at 10 °C. In contrast, at 20 °C, the same effect was seen under all conditions, with a sharp increase in activity at medium light irradiation. Similarly, identical effect was observed at 30 °C under all conditions, with the highest activity at the highest light irradiation (Fig. 7b). Significantly (*p* < 0.05), the highest SOD activity (17.77 ± 2.63 U mg^−1^ protein) was observed when cells were grown with low or medium nitrogen (0.05–0.5 mM KNO_3_) at 20 °C under light irradiation of 145 µmol photons m^−2^ s^−1^ compared to all combination of light, temperature, and nitrogen concentration.

#### POD Activity

POD activity increased with light irradiation when cells were grown at all three temperatures and with all three nitrogen levels except when cells were cultured with 0.5 mM KNO_3_ at low temperature under medium light irradiation (Fig. 7c). Cells cultured with 0.5 mM KNO_3_ at 10 °C under medium light resulted in maximum POD activity (0.084 ± 0.011 U mg^−1^ protein). A three-way ANOVA indicates that light intensity (*p* < 0.001) and nitrogen concentration (*p* = 0.01) are significantly affecting POD activity in cells. However, temperature (*p* = 0.050) is not statistically significant. The interactions of light × temperature (*p* < 0.001), light × nitrogen (*p* < 0.001), temperature × nitrogen (*p* = 0.007), and light × temperature × nitrogen (*p* < 0.001) all have a significant effect on POD activity. POD activity decreased with increase in nitrogen concentration in cells when cultured under high light irradiation, but the opposite scenario was observed when cells were grown under low light irradiation at the same temperature (Fig. 7c). Significantly (*p* < 0.05) higher POD activity was observed when cells were grown with low nitrogen (< 0.5 mM KNO_3_) at 10 or 20 °C under high light irradiation (550 µmol photons m^−2^ s^−1^) relative to cells grown with high nitrogen under the same light and temperature. Though POD activity did not significantly change with the variation of nitrogen concentrations when cells cultured at 30 °C under > 145 µmol photons m^−2^ s^−1^, it was significantly (*p* < 0.05) higher in cells grown with high nitrogen under low light irradiation relative to cells cultured with limited nitrogen (< 0.5 mM KNO_3_) under the same temperature and light irradiation (Fig. 7c).

From the above SPSS (data analysis), optimal level of factors cannot be selected to obtain the highest level of antioxidant content; therefore, design of experiments (Minitab Statistical Software) was further used. Design of experiments (Minitab Statistical Software) predicted the best combination of culturing factors levels for the highest accumulation of enzymatic and non-enzymatic antioxidants in *Dunaliella tertiolecta* are shown in Table 2.

**Table 2:** DoE optimised best combination of culturing factors compared with literature reported levels of antioxidant components

Intracellular components in *D. tertiolecta* in response to variation of light, temperature, and nitrogen concentration during cultivation (this study)			Intracellular components in *Dunaliella* in response to variation of light, temperature, and nitrogen concentration during cultivation		
Compounds	Optimal levels	Response (maximum production)	Compounds	Culture conditions	Reference
Chlorophyll (µg 10^−7^ cells)	L (15) + T (20) + N (5)	64.28	36.5 ± 6.1	L (200) + T (25)	(Xu et al. 2016)
			16	L (100) + T (28)	(Haghjou et al. 2009)
			7	L (100) + T (28)	
			14.28	T (28) + N (250)	(Singh et al. 2016)
			20.45	T (37) + N (0.75)	
			20	L (190) + N (5)	(Gallego-Cartagena et al. 2019)
			70 ± 11	L (60) + (N + S) (starvation)	(Lv et al. 2016)
			100	L (68) + T (22–30)	(Wu et al. 2016)
Carotenoid (µg 10^−7^ cells)	L (15) + T (20) + N (5)	10.49	8.1 ± 1.1	L (50) + T (25)	(Xu et al. 2016)
			6.8	T (37) + N (0.75)	(Singh et al. 2016)
			3.5	T (28) + N (250)	
			10	L (190) + N (5)	(Gallego-Cartagena et al. 2019)
			29	L (390) + N (0.5) + NaCl (4 M)	
			250	L (800)	(Nguyen et al. 2016)
Total protein content (mg 10^−7^ cells)	L (15) + T (10) + N (5)	1.40	0.266 ± 0.04	L (1000) + T (25)	(Xu et al. 2016)
			0.62 ± 0.12	L (150) + N (5)	(Yilancioglu et al. 2014)
			0.10	L (100) + T (28)	(Haghjou et al. 2009)
			0.27	L (100) + T (28)	
Non-enzymatic antioxidant					
Phenolic compounds (µg GAE 10^−7^ cells)	L (15) + T (20) + N (0.5)	39.59			
Total ascorbate (nmol 10^−7^ cells)	L (550) + T (20) + N (0.5)	200	18	L (100) + T (13)	(Haghjou et al. 2009)
			14	L (1200) + T (28)	
Total glutathione (nmol 10^−7^ cells)	L (550) + T (20) + N (0.05)	39.67	3	L (1200) + T (13)	(Haghjou et al. 2009)
			2	L (1200) + T (13)	
Enzymatic antioxidant					
CAT activity (U mg^−1^ protein)	L (15) + T (NS) + N (5)	14.34	6.67 ± 0.89	L (150) + 3 M NaCl	(Jahnke and White 2003)
			0.12 ± 0.01	L (150) + N (5)	(Yilancioglu et al. 2014)
			20.13	UV + N (optimum)	(Tian and Yu 2009)
			31.45	16% NaCl + N (5)	
SOD activity (U mg^−1^ protein)	L (145) + T (20) + N (NS)	15.19	24.8 ± 2.63	UV + N (optimum)	(Al-Rashed et al. 2016)
			14.25 ± 0.45	UV + N (Starvation)	
			20.36	UV + N (optimum)	(Tian and Yu 2009)
			33.61	2.8 M NaCl + N (5)	
			0.625 ± 0.09	L (150) + 3 M NaCl	(Jahnke and White 2003)
POD activity (U mg^−1^ protein)	L (145) + T (10) + N (0.5)	0.083			

Symbol L, T, N, NS indicate the light, temperature, nitrogen levels, and not significant, respectively. The unit of light is presented as µmol photons m^−2^ s^−1^, temperature as °C, and nitrogen level as mM KNO_3_

## Discussion

The combined effect of light, temperature, and nitrogen levels in the cultivation medium was used to induce stress, and both enzymatic and non-enzymatic antioxidant levels in *D. tertiolecta* were quantified. For the development of a deeper understanding of the overall intracellular antioxidant response, the accumulation of chlorophyll and carotenoid content, cell density, total protein, H_2_O_2_, and MDA content were also quantified. SOD provides the first-line defence against toxic effects of ROS by catalysing the dismutation of O_2_^·−^ to H_2_O_2_ and O_2_. CAT directly converts H_2_O_2_ into H_2_O and O_2_, whereas POD scavenges H_2_O_2_ by utilising ascorbate or glutathione as an electron donor (Chokshi et al. 2017).

Predictably, cells grown at both high nitrogen under medium to high light irradiation exhibited highest cell density (Fig. 1a), whilst less antioxidant activity were detected in cells grown with both high light and high nitrogen (Figs. 5 and 6). At high light irradiation, the chloroplastic pigments of the cell will more efficiently absorb energy, which enhances the conversion rate of chemical energy into photochemical reactions (Gu et al. 2014) resulting in a higher growth rate. The observed lower cell density at a low nitrogen level (0.05 mM) and low temperature might be due to the reduction of nutrient assimilation (Ras et al. 2013), lower production of chlorophyll and chloroplastic proteins (Fig. 1b), which in turn decreases the cells light absorption capacity (Benavente-valdes et al. 2016) resulting in a lower the growth rate and the generation of oxidative stress. Therefore, lower antioxidant production and maximum biomass can be obtained in *D. tertiolecta* cultures, when cells are grown at 20 °C under 145 µmol photons m^−2^ s^−1^ or higher in the presence of high nitrogen concentration (5 mM KNO_3_), which would be the optimal growth conditions. This result is in agreement with the study of Wu et al. (2016), who reported a temperature of 20 °C and light irradiation of 135 µmol photons m^−2^ s^−1^ for optimal *D. salina* growth.

Total protein content also substantially increased at high nitrogen concentration in all combinations of factors (Fig. 2), as nitrogen is essential for the formation of structural and functional peptides, proteins and enzymes (Cai et al. 2013). A positive correlation between nitrogen concentration and protein accumulation in three *Chlorella* strains was reported in the study by Ordog et al. (2016), which is in agreement with the result presented here. During nitrogen starvation, carbon flow is directed from protein synthesis to fatty acid and carbohydrate synthesis (Yilancioglu et al. 2014); causing a reduced protein production (Fig. 2). Moreover, during nitrogen deprivation proteins may degrade to sustain short-term growth, resulting in decreased protein content (Ördög et al. 2016). The result (Fig. 2 and Table 2) indicates that low temperature along with low light culture conditions are favourable for protein accumulation in *D. tertiolecta*, which may be due to carbon from polysaccharides, chlorophyll a, and photosynthate incorporated to protein synthesis (Rivkin 1989). The low chlorophyll content (per cell) (Fig. 1b) in cells cultivated at low temperature with low light irradiation indicates that chlorophyll may be involve in the synthesis of protein. Thompson et al. (1992) also reported an increased protein accumulation in *D. tertiolecta* as the temperature dropped from 15 to 10 °C. Similarly, higher protein content was reported in *P. tricornutum* when growth temperature reduced from 18 to 7 °C. Since antioxidant enzymes (CAT, POD, and SOD) are proteinous molecules, it is expected that these enzymes will be stimulated with the changes of culture conditions.

The highest CAT activity was found in cells when grown with high nitrogen under low light irradiation, whilst POD activity was maximum under medium light irradiation (Fig. 7a, c and Table 2). Nitrogen availability increases the rate of photosynthesis and the number of electrons transported through the Z-scheme electron pathway (Li et al. 2016), which further increases the photorespiration rate and other metabolism and produces H_2_O_2_ (Zervoudakis et al. 2015) resulting in an increased level of CAT activity. The low H_2_O_2_ content in cells grown with high nitrogen at low temperature and under low light irradiation (Fig. 3a) could indicate maximum detoxification of H_2_O_2_ (highest stimulation of CAT activity). The predomination of CAT in eliminating H_2_O_2_ at low temperature may decrease POD response (Chokshi et al. 2017) as CAT has a high turnover rate (one molecule can convert 6 million H_2_O_2_ to H_2_O and O_2_ within one minute) (Gill and Tuteja 2010). Low light irradiation reduces the rate of carbon assimilation and the photosynthetic apparatus is rearranged in order to increase light absorption efficiency, which diverts carbon metabolism from lipid biosynthesis toward the synthesis of the light-harvesting antenna protein complexes (Falkowski et al. 1981; Sukenik et al. 1990). This unbalanced C-N metabolism produces oxidative stress (Zhang et al. 2010), stimulating CAT activity.

The accumulation of H_2_O_2_ and MDA content differs in response to variation in light, temperature, and nitrogen concentration during cultivation of *D. tertiolecta* (Fig. 3a, b). These components are all used as oxidative stress markers (Chokshi et al. 2017; Zhu et al. 2017). The highest H_2_O_2_ content simultaneous with the highest CAT and POD activity, and the lowest MDA content together with the highest SOD activity at high temperature when cells grown with high nitrogen and low light irradiation (Table 2) demonstrate that oxidative stress is eliminated by SOD by converting O_2_^−·^ into H_2_O_2_, which further is reduced by both CAT and POD (Figs. 3 and 7). A decrease in the reduced form of ascorbate and glutathione also indicates the use of these components as a substrate (Sharma et al. 2012) in the peroxidase reaction processes (Figs. 5 and 6). Accumulated reduced ascorbate and glutathione content in cells grown under low light irradiation indicated that these components act as antioxidants to prevent cells damage from the oxidation stress generated by low light (Fig. 5). The higher ratio of ASc:DASc in cells indicate the enhancement of the reduced form of ascorbate, which occur not only at the expense of its oxidised form (DASc) reduction but also due to de novo molecule synthesis (biosynthesis from different molecules) (Radyuk et al. 2009). Accumulation of pigments (chlorophyll and carotenoid) was highest in *D. tertiolecta* cells when grown under low light irradiation accompany with high nitrogen concentration (Fig. 1a–c and Table 2), which suggest that these are involved in protecting cells from low light-induced oxidative stress. The chlorophyll content is directly linked to the cells photosynthetic capacity (Zhu et al. 2017). In contrast, carotenoids protect cells from photodamage by scavenging singlet oxygen and other reactive oxygen species and by absorbing heat (Yilancioglu et al. 2014); therefore, to maximise light energy absorption at low light irradiation, pigment content increases (Beneragama and Goto 2010; Robinson et al. 1995)*.*

Though CAT activity was found to be at its highest when cells were grown with high nitrogen, POD and SOD activity substantially increased at limited nitrogen concentration and high light irradiation (≥ 145 µmol photons m^−2^ s^−1^), which indicates that POD and SOD activity is highly responsive to high light irradiation and low nitrogen. Accumulation of H_2_O_2_ and MDA content (Fig. 3a, b) simultaneous with higher POD and SOD activity at low temperature when cells were grown under high light irradiation demonstrates that oxidative stress was mitigated by POD combined with SOD. High light may cause overexpression of the SOD gene (Fe-SOD) in *D. salina* (Park et al. 2006), and may bring about an increased flow of electrons in the electron transport chains of the photosystem, thereby leaking more electrons onto O_2_, generating superoxide ion (O_2_^**−.**^). Cells grown with nitrogen concentration (≤ 0.5 mM KNO_3_) under high light irradiation also enhanced the accumulation of total glutathione and total ascorbate content (Fig. 5), which may be a way to mitigate high light and low nitrogen-induced oxidative stress (Wang et al. 2010). An increased level of reduced ascorbate content under high light coupled with nitrogen level (0.5 mM KNO_3_) indicates the role of ascorbate as a cofactor of violaxanthin de-epoxidase, thus, sustaining dissipation of excess excitation energy and may directly react with ROS (O_2_^•−^, H_2_O_2_) to protect against cell damage (Sharma et al. 2012). The findings presented here are in agreement with the results of Haghjou et al. (2009), who observed increased glutathione, ascorbate, SOD and POD activity in *D. salina* when these cells were grown at low temperature under high light irradiation (Table 2). Similarly, an increased glutathione content with increases in light irradiance levels was observed in an endosymbiotic dinoflagellate (*Symbiodinium*) (Muhaemin et al. 2017), which is also consistent to the findings presented here. In addition, the result is in line with the investigation of El-Baky et al. (2004), who obtained high POD and SOD activity as well as high total glutathione and ascorbate content in limited nitrogen cultures of *D. salina* under high light irradiation*.* Al-rashed et al. (2016) and Lv et al. (2016) also reported that *D. salina* subjected to nitrogen starvation-induced oxidative stress increased SOD activity. Compared to low light, chlorophyll and carotenoid content were reduced under high light (Fig. 1b, c), which may be due to damage of the photosystems centre (PSII) and reduced levels of the light-harvesting apoproteins in the chloroplast thylakoids in the cells (Neidhardt et al. 1998; Sukenik et al. 1990). However, high cell density was found when the cultures were grown with high nitrogen levels under high light irradiation, which may be due to this algae’s efficient repair cycle that allows it to replace damaged PSII at a much faster rate to maintain maximum photosynthetic efficiency (Gu et al. 2014; Xu et al. 2016). A study of Xu et al. (2016) reported an increase in optical density with a decreased chlorophyll and carotenoid content in *D. tertiolecta* under high light irradiation, which is consistent to the presented finding.

Compared to cells grown at 20 °C with low nitrogen under low light irradiation, the accumulation of MDA and SOD activity in cells when grown under high light irradiation with the same temperature and nitrogen indicates the inversely proportional relationship of MDA to SOD; SOD could not eliminate superoxide radicals from the cells. Therefore, glutathione or ascorbate participated in protecting cells from high light and low nitrogen-induced stress. Total phenolic content was found to be its highest when cells grown at 20 °C with low nitrogen and low light irradiation (Fig. 2b and Table 2) demonstrating that phenolic compounds take part in mitigating low nitrogen and low light-induced stress (Ismaiel 2016) and act as an antioxidant. Phenolic biosynthesis could occur as an alternative pathway for photochemical energy dissipation enabled to enhance the antioxidant capacity of the cell (Grace and Logan 2000) under stress conditions, whilst carbon flow in the phenolic biosynthesis is usually through the phenylpropanoid pathway (Kepekci and Saygideger 2012) during optimal growth condition. The result is in agreement with a study by Aremu et al. (2015), who demonstrated that moderate nitrogen concentration promoted phenolic content in *Chlorella minutissima* (Aremu et al. 2015). A study by Kepekci and Saygideger (2012) reported the lowest phenolic content in *Spirulina platensis* cells when cultured at 30 °C under 40 μmol photons m^−2^ s^−1^, whilst the highest amount was found in the cultures incubated under 120 μmol photons m^−2^ s^−1^, which is opposite to the findings presented here. This might be due to variability between strains. A study of Al-Rashed et al. (2016) reported that nitrogen deprivation enhanced total phenolic in two strains (*D. salina* and *Spirulina platensis*), which is consistent with this study. As there are limited studies available on the accumulation of phenolic compounds by microalgae, more research is still needed to determine how phenolic content responds to the accumulation of ROS in different microalgae under various stress conditions.

As limited nitrogen availability under low light irradiation in cultures stimulated ascorbate, glutathione, and phenolic contents, these components collectively mitigate low light and low nitrogen-induced oxidative stress. Nitrogen and temperature did not have a significant impact on the accumulation of phenolic compounds when cells were grown at medium light irradiation (145 μmol photons m^−2^ s^−1^). However, glutathione content was the lowest in cells when grown under medium light irradiation together with all combinations of nitrogen and temperatures indicating that glutathione may directly scavenge free radical (O_2_^•−^, ^•^OH, H_2_O_2_) or can participate in the regeneration of another potential antioxidant ASc, via the ASc-GSH cycle (Sharma et al. 2012). The highest POD activity (Table 2) and the lowest ascorbate levels were found in cells when grown at low temperature with moderate nitrogen (0.5 mM KNO_3_) under medium light irradiation which indicates the predomination of POD to minimise H_2_O_2_ content relative to CAT. This result is consistent with a study by Haghjou et al. (2006), who reported high POD activity at 13 °C under light irradiation (100 µmol photons m^−2^ s^−1^) in *D. salina* cell cultures.

**Fig. 8:**
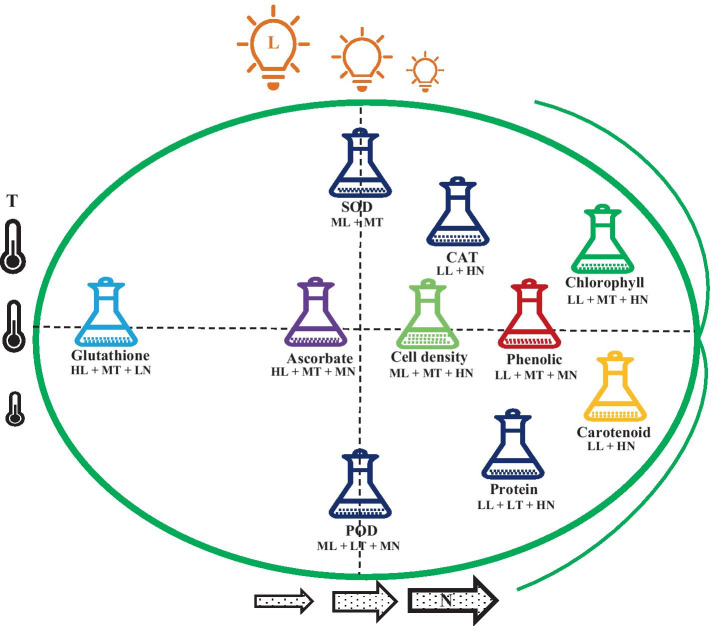
Visual presentation of the effect of light, temperature, and nitrogen levels on cellular antioxidant system in *D. tertiolecta* (represented by L, T, and N, respectively). LL, 15; ML, 145; HL, 550 µmol photons m^−2^ s^−1^; LT, 10; MT, 20; HT, 30 °C; LN, 0.05; MN, 0.5; HN, 5 mM)

Both chlorophyll and carotenoids achieved their highest level in cells when grown at a medium temperature under low light irradiation and high nitrogen levels (Table 2). Protein content and CAT activity are both highly responsive to stress induced by low light irradiation combined with a high nitrogen content with temperature also being significant for protein content. Total phenolic content was found to be highest when cells were grown with medium nitrogen at medium temperature and low light irradiation. Both POD and SOD activities were maximum in cells when grown under the medium light irradiation, but the highest POD activity was found at low temperature with medium level of nitrogen and SOD activity at medium level of nitrogen. Total ascorbate and glutathione content reached its highest levels in *D. tertiolecta* when cells were grown with limited nitrogen under high light irradiation and medium temperature (Fig. 8 and Table 2).

## Conclusion

In this study, antioxidant responses of *D. tertiolcta* cells exposed to different levels of light, temperature, and nitrogen were investigated. The results demonstrate that high cell density in *D. tertiolecta* can be obtained in culture when cells were grown with high nitrogen at medium temperature under medium light irradiation. The accumulation of chlorophyll and carotenoid was maximum in cells grown with high nitrogen levels under low light irradiation at a medium temperature. Similarly, maximum total protein and CAT activity were found at the same light and nitrogen levels; however, temperature has no significant impact on CAT activity. The highest phenolic content in *D. tertiolecta* cells was observed when cells were grown with medium nitrogen level at a medium temperature under low light irradiation. Cells grown with medium nitrogen at a medium temperature under high light irradiation accumulated maximum total ascorbate content. Similarly, total glutathione content was maximum when cells were grown under the same light and temperature, but in the presence of low nitrogen content in the culture. Maximum SOD activity in *D. tertiolecta* was found when cells were cultured under medium light irradiation at a medium temperature. However, the highest POD activity was achieved in cells grown with medium nitrogen under medium light irradiation at low temperature. Optimal values indicate that a further experiment is warranted as responses possible would increase if conditions are extended beyond this research range or investigated in a narrower range.

These results provide the overall physiological responses, including antioxidant enzymes activity in *D. tertiolecta* cells under three levels of light, temperature, and nitrogen concentration, and indicate a novel strategy to improve the antioxidant production. Also, scientific information obtained from this study on the production of enzymatic and non-enzymatic antioxidants in *D. tertiolecta* has ‘opened the door’ to additional possibilities for commercial exploitation.
